# Antibacterial Activities of Five Medicinal Plants in Ethiopia against Some Human and Animal Pathogens

**DOI:** 10.1155/2018/2950758

**Published:** 2018-01-11

**Authors:** Gebremedhin Romha, Birhanu Admasu, Tsegaye Hiwot Gebrekidan, Hailelule Aleme, Gebreyohans Gebru

**Affiliations:** ^1^Department of Animal Production and Technology, College of Agriculture and Environmental Science, Adigrat University, Adigrat, Ethiopia; ^2^Department of Animal and Range Science, College of Agriculture and Natural Resources, Dilla University, Dilla, Ethiopia; ^3^Department of Chemistry, College of Natural and Computational Science, Dilla University, Dilla, Ethiopia; ^4^Department of Animal and Range Science, Dilla University, Dilla, Ethiopia; ^5^Department of Animal Sciences, College of Agriculture, Aksum University, Shire Campus, Shire, Ethiopia

## Abstract

**Objective:**

To evaluate the* in vitro* antibacterial activities of five plant extracts which have been used as traditional medicines by local healers against three multidrug resistant bacteria, namely,* Staphylococcus aureus*,* Escherichia coli*, and* Pseudomonas aeruginosa*.

**Results:**

The highest mean zone of inhibition (4.66 mm) was recorded from methanol extract of* Calpurnia aurea* (Ait.) Benth. at a concentration of 200 mg/ml against* S. aureus*, followed by* Croton macrostachyus *Del. (4.43 mm) at the same dose and solvent for the same bacterial species, while methanol and chloroform extracts of* E. brucei* Schwein. did not inhibit growth of any bacterial species. The lowest value (100 *μ*g/ml) of minimum inhibitory concentration (MIC) was observed from both methanol and chloroform extracts of* C. aurea* (Ait.) Benth. against all the three bacteria. The results of the positive control had no statistically significant difference (*P* > 0.05) when compared with crude extracts of* C. aurea* (Ait.) Benth. at concentration of 200 mg/ml against* S. aureus*.

**Conclusion:**

The results of the present study support the traditional uses of these medicinal plants by the local healers. Except* Erythrina brucei* Schwein., all the plants investigated in this study exhibited antibacterial activities against the test bacterial species. Further researches are needed to be conducted to evaluate efficacy of these medicinal plant species on other microbes in different agroecological settings and their safety levels as well as their phytochemical compositions.

## 1. Introduction

Although plenty of guidelines have been developed and set for application to prevent foodborne diseases and food spoiling microorganisms, it is impossible totally to be free of them.* S. aureus* and* E. coli* are among the major causes of foodborne diseases [1, 2]. Species of* Pseudomonas *are also predominant causes of food spoilage [3, 4]. The emergence of drug resistance to multiple antibiotics worsens the problem. Nowadays, reports have indicated that* Staphylococcus aureus (S. aureus)*,* Escherichia coli (E. coli),* and* Pseudomonas aeruginosa (P. aeruginosa)* are not only multidrug resistant pathogens [5–7] but also broadly drug-resistant and pandrug-resistant bacteria [8]. For instance, it has been reported worldwide that* S. aureus* isolates are increasingly resistant to a greater number of antimicrobial agents as reviewed in Lowy [5]. Multidrug resistance in* E. coli* increased from 7.2% during the 1950s to 63.6% during the 2000s, and particularly considerable growing trend in resistance was noticed for ampicillin, sulfonamide, and tetracycline [6].* P. aeruginosa* has also showed resistance to the most frequently administered antipseudomonal antibiotics: *β*-lactams, aminoglycosides, and fluoroquinolones [7].* S. aureus*,* E. coli*, and* P. aeruginosa *are also responsible for the majority of nosocomial infections [9].

Antibiotics that show low efficacy in treating human and animal diseases through antibiotic resistance must be replaced with new drugs to combat the burden of these pathogens [10]. Hence, medicinal plants are expected to be the best source of obtaining a variety of drugs [11, 12]. In Ethiopia, different communities have extensively been using medicinal plants to treat different diseases for many centuries [13–15]. Such plants, however, need to be investigated for better understanding of their properties, safety, and efficiency [16]. Medicinal plants including* C. aurea *(Ait.) Benth.,* C. macrostachyus* Del., and* Withania somnifera (W. somnifera)* (L.) Dunal have been tested for their antimicrobial efficacy* in vitro* and* in vivo* in different countries [17–25]. Previous information of the current study plants is presented in Table 1. However, testing the efficacy of these plants in different agroecological zones can make the evidence more strong since agroecology (e.g., geographic location and soil type) could affect the accumulation of bioactive pharmaceutical ingredients that are found in plants [26].

As reviewed in Mahidol et al. [27], medicinal plants have been used for treating infectious diseases because of their ease of accessibility as well as their lower side effects and toxicity. Medicinal plants may provide an exceptional renewable resource for the discovery of potential novel pharmaceuticals as their constituents have ample structural and biological diversity [27–29]. In Ethiopia, many plant species are being used as traditional medicine for the treatment of various human and animal diseases [20, 30–39]. Hence, more studies related to the use of medicinal plants as remedial agents need to be conducted, especially for plants which are useful to control antibiotic resistant microbes [40]. The aim of this study was to evaluate the efficacy of five plant extracts, which have been used as traditional medicines by local healers for their* in vitro* antibacterial activities against three multidrug resistant bacteria, namely,* S. aureus*,* E. coli*, and* P. aeruginosa*.

## 2. Materials and Methods

### 2.1. Preparation of Plant Material and Crude Extracts

Five species of plants (Table 2), which have been widely used by local healers in different areas of southern Ethiopia for the treatment of livestock diseases [39], were collected from different agroecological zones to evaluate their* in vitro* antibacterial effects. The study was conducted from December 2015 to September 2016 at Dilla University.* C. aurea* (Ait.) Benth. and* W. somnifera* (L.) Dunal were collected from Yabello district, Borena zone, Oromia regional state, which is 5-6 km far from Yabello town.* C. macrostachyus *Del., and* E. brucei *Schwein. were collected from Dilla University Campus, Gedeo zone, while* N. tabacum *L. was collected from Seid Hassen Integrated Farm (private farm), Sidama zone, which is located at around 30 km west of Dilla town. Plant leaves and roots (for* W. somnifera* (L.) Dunal) were collected from their natural habitat and washed with tap water to remove unnecessary particles and dried under shade at a room temperature for about a month.

The procedure used for the preparation of the plant materials was adapted from Eloff [46]. Briefly, the dried plant materials were then ground using a commercial blender. The powders of each plant were weighed using precision standard electronic balance before maceration (Table 2). Maceration of each plant was carried out in flasks containing methanol (99.8%) and chloroform (95%) separately in a 1 : 10 (solute : solvents) ratio enough to cover all the plant powders with a continuous shaking with an orbital shaker (Gemmy Industrial Corporation, VRN-480) at 100 rpm and an occasional manual stirring with a glass rod. After 80 hours, the macerates of each plant were filtered in separate flasks using a qualitative filter paper (what man 44). The residues after filtration process were discarded while the filtrate parts were taken to a vacuum rotary evaporator (RE-52A, 220 V/50 Hz) machine for extraction with 35–40°C temperature adjustment. The methanol and chloroform crude extract products were poured into evaporating dishes and kept in dry oven (DHG-9030) at 40°C for 3-4 days until the extract materials were concentrated. Finally, the percentage yield of each plant extract was calculated (Table 2).

### 2.2. Agar Well Diffusion and MICs

Media preparation and agar well diffusion assay were done as previously described [46]. The American Type Culture Collection of* Staphylococcus aureus* 25923,* Pseudomonas aeruginosa* 27853, and* Escherichia coli* 20922 were obtained from the Ethiopian public health institute and were maintained on Brain Heart Infusion (BHI) agar medium (HiMedia) at 4°C until the commencement of the experiment. The test suspension was standardized to match 0.5 McFarland turbidity standard which corresponds to approximately 1.5 × 10^8^ CFU/ml. A working solution (50 *μ*g/ml) of streptomycin (mM EDTA, analytical standard) was prepared from stock solution as a positive control, and 5% DiMethyl Sulphoxide (DMSO) (ACS reagent grade) was used as a negative control.

Three concentrations, 200, 100, and 50 mg/ml, of 5 crude plant extracts were used as a test material. Standardized inocula were swabbed uniformly onto solidified Mueller Hinton agar and the seeded media were allowed to dry for about 5 minutes. Five wells per plate (three wells for extracts, one for each of the positive and negative control) were made in the seeded agar using 6 mm cork borer. With the help of micropipette, 20 *μ*l of test solution was poured into the respective wells. The media were kept in the biosafety for 1 hr for proper diffusion and thereafter incubated at 37°C for 24 hrs. The experiment was repeated three times and the results, zones of inhibition of each dose, were expressed as the mean of the replicates.

The MIC was evaluated using agar dilution method [46], and MHA culture media were used. Serial dilutions of the plant extracts were prepared by dissolving in 5% DMSO to make concentrations of 15.625–1000 *μ*g/ml. Two ml aliquot of the plant extract dilution of each concentration to be tested was added to 19 ml of the molten agar. The test was performed in triplicate.

### 2.3. Data Management

Data were entered into Microsoft Excel, 2010, and were transformed using the square root to maintain the normal distribution among the harvested data. Data analysis was performed using JMP statistical software, version 5 (USA, 2002). The model employed was full-factorial ANOVA and expressed with the equation stated below.

(1) $$\begin{array}{c} y=\mu +a+b+c+ab+ac+bc+abc+\varepsilon , \end{array}$$

where *y* represents the overall response; *a*, *b*, and *c* stand for plant species, dose, and bacterial species, respectively, while *μ* and *ɛ* represent overall mean and common error, respectively. *ab*, *ac*, *bc*, and *abc* indicate the interaction effects of the respective variables. Zone of inhibitions were expressed as mean. Whenever significant variation was detected among treatments, means were separated using Tukey HSD at 5% level of significance.

## 3. Results

### 3.1. Percentage Yield of Plant Extracts

Five medicinal plants prioritized by Romha et al. [39] based on their preference by local healers for treating animal and human diseases were collected and extracted (see Materials and Methods) using two solvents: methanol and chloroform. The highest (21.3%) and lowest (9.3%) yields were harvested from chloroform extracts of* W. somnifera* (L.) Dunal and methanol extracts of* N. tabacum *L., respectively (Table 2).

### 3.2. Antibacterial Activities of Plant Extracts

The methanol and chloroform extracts of the plant species listed in Table 2 were tested using different concentrations (50, 100, and 200 mg/ml) against three multidrug resistant bacterial species:* S. aureus, P. aeruginosa,* and* E. coli*. Of all the plants tested,* C. aurea* (Ait.) Benth. showed a wide spectrum of antibacterial activities against the investigated bacterial species. Regarding the dosage and solvents, 200 mg/ml showed best antibacterial activities both in solvents and in all plant species. Better antibacterial activities with maximum zone of inhibition (4.66 mm) in* C. aurea *(Ait.) Benth. were recorded in methanol crude extracts at a concentration of 200 mg/ml against* S. aureus* followed by* C. macrostachyus* Del. (4.43 mm) at the same dose and solvent for the same bacterial species (Table 3).

Moreover, good results were observed from methanol and chloroform extracts of* C. macrostachyus* Del. against* P. aeruginosa*,* E. coli,* and* S. aureus *at a concentration of 200 mg/ml. Methanol extracts of* W. somnifera* (L.) Dunal showed comparable effect on these bacterial species at the same dose. Methanol and chloroform extracts of* E. brucei* Schwein. did not inhibit the growth of any bacterial species (Tables 3 and 4). The MIC for crude extracts of each plant for the respective test bacterium was done using the agar dilution method. The lowest value (62.5 *μ*g/ml) of MIC was recorded from both methanol and chloroform extracts of* C. aurea* (Ait.) Benth. against all the test bacteria. For* C. macrostachyus* Del.,* W. somnifera* (L.) Dunal, and* N. tabacum* L., the MICs were between 250 *μ*g/ml and 1000 *μ*g/ml in all the test bacteria in both extractants.* E. brucei* Schwein. did not inhibit the growth of all bacteria employed in this study.

## 4. Discussion

### 4.1. The* In Vitro* Efficacy of Plant Extracts

#### 4.1.1. *Calpurnia aurea* (Ait.) Benth.

The selected medicinal plant species (Table 2) commonly reported by local healers [39] were evaluated for their efficacy* in vitro* against three bacteria species:* S. aureus, P. aeruginosa,* and* E. coli*. Different zones of inhibition were recorded from each plant extract and from different concentrations for each species of bacterium. The highest zones of inhibition (4.66 mm) were observed from* C. aurea* (Ait.) Benth. at concentration of 200 mg/ml against* S. aureus* from methanol extract which had no statistically significant difference (*P* > 0.05) when compared with the positive control. Consistent with the present study, comparable zones of inhibition with the positive control were reported from methanol extracts of* C. aurea* against* S. aureus, P. aeruginosa,* and* E. coli* in South Africa [17]. Promising antibacterial activities of methanol extracts of* C. aurea* on these bacterial species have been reported in Ethiopia [21]. Antihypertensive [41], antioxidant [17], and antiparasitic [47] activities of* C. aurea* have also been reported.

#### 4.1.2. *Croton macrostachyus* Del.

Promising antibacterial activities were obtained from methanol and chloroform leaf extracts of* Croton macrostachyus* Del. against the test bacteria at a concentration of 200 mg/ml. The best activity, even more than the positive control, has been reported from methanol extract of* C. macrostachyus* against* S. aureus* in a previous study conducted in Ethiopia [20]. The methanol extracts of* C. macrostachyus* Del. also showed a potential antibacterial activity against* E. coli* and* P. aeruginosa* [18]. An interesting activity has been recorded from methanol extracts of leaf* C. macrostachyus* against wide range of Gram-positive and Gram-negative bacterial species including* S*.* aureus, P. aeruginosa,* and* E. coli* [25]. Moderate antibacterial activity result has also been recorded from stem bark extract of* C. macrostachyus* [24]. In fact, antibacterial secondary metabolites such as polyphenols, triterpenes, and saponins were isolated from the leaf of this plant [25]. Moreover, antimalarial [22] and insecticidal [44] activities of leaf extracts of this plant have also been reported in Ethiopia.

#### 4.1.3. *Withania somnifera* (L.) Dunal

Though* W. somnifera* (L.) Dunal has been used by the Ethiopian local healers for the treatment of different animal and human ailments [34–37, 39], scientific data on its antimicrobial activity is inadequate. Methanol extract of* W. somnifera *(L.) Dunal (root) revealed a promising antimicrobial activity against the test bacteria while chloroform extract of the plant showed moderate activity.* W. somnifera* is widely used as traditional medicine in Pakistan and strong antibacterial activities have been reported from methanol extract of* W. somnifera *(leaf) in the same country as reviewed in Adnan et al. [48]. High and moderate antibacterial activities have been recorded for methanol extract of* W. somnifera* (L.) Dunal stem against* E. coli* and* P. aeruginosa*, respectively, in India [23]. Leaves, fruits, and roots of* W. somnifera* showed the same antibacterial activities against these two bacterial species and the highest activities were recorded from the plants' leaves [19].* W. somnifera* also exhibited antioxidant [19] and anti-inflammatory [49] properties.

#### 4.1.4. *Nicotiana tabacum* L. and* Erythrina brucei* Schwein.

Weak antimicrobial activities were observed from methanol and chloroform extracts of* N. tabacum* L. against all the test bacteria. Moreover,* E. brucei* Schwein. did not totally inhibit the bacterial growth in the present study. However, good antimicrobial activities from methanol extract of* N. tabacum* L. have been reported from a previous study [20]. The difference in its medicinal value may possibly be risen due to agroecological difference. The use of these plants as a traditional medicine to treat various diseases of humans and animals is commonly reported in Ethiopia [20, 33, 36, 38, 39] and in Pakistan [50].* E. brucei* Schwein. did not inhibit the bacterial growth in all concentrations. The plant might be active against parasites which supports the traditional belief documented previously [39]. However, this is preliminary evidence and needs further researches. Additional researches on antimicrobial activities as well as their phytochemical analysis of these plants should be conducted.

### 4.2. Sensitivity of the Test Bacteria to the Extracts

The highest zone of inhibition was recorded in the crude extract tested against* S. aureus. *Previous experimental works showed that, among a group of bacteria tested for crude extracts of different plant species,* S. aureus* was found sensitive comparably in all plant extracts [20, 51–53]. Likewise, considerable studies indicated that extracts from different plant species exhibited better activities on Gram-positive bacteria than on Gram-negative bacteria [54–61], while some studies reported that* P. aeruginosa* [61, 62] and* E. coli* [21] were also sensitive to extracts of some medicinal plants. These sensitivity differences between Gram-positive and Gram-negative bacteria to the extract of different medicinal plants might be due to the structural and compositional differences in membranes between the two groups [63]. Indeed, Gram-negative bacteria are more resistant to antibiotics because they possess impermeable outer membrane; consequently, the levels of antibiotics in the cell are reduced [64].

Previous study indicated that plant extracts with MICs up to 100 *μ*g/ml have significant antibacterial activity. If the MICs of the plant extracts are between 100 and 625 *μ*g/ml and above 625 *μ*g/ml, the antibacterial activity of the respective plant extract is considered to be moderate and low, respectively [65]. The present study had its own limitation in that the method we employed for testing the plants' crude extracts was agar well diffusion; thus, nonpolar compounds may not diffuse well into the aqueous agar matrix thereby antibacterial activity of the plants may be underestimated.

## 5. Conclusion

The results of the present study support the traditional uses of these plants practiced by the local healers. Except* E. brucei* Schwein., all the plants investigated in this study exhibited antibacterial activities against the test bacterial species. However, it was reported that* E. brucei* Schwein. has been used for the treatment of external and internal parasites [39]. Further researches are needed to be conducted to evaluate the efficacy of these medicinal plant species on other microbes in different agroecological settings and their safety levels as well as their phytochemical compositions.

## Figures and Tables

**Table 1:** Previous information of the present study plants.

Plant name: species/family/voucher number	Reported traditional use (veterinary or human)	Parts used	Previously screened activities	Country/reference
*C. aurea* (Ait.) Benth./Fabaceae/NA	Human: diarrhea, dysentery, and stomach disorder	Leaves and fruit		Ethiopia/[31]

*C. aurea/*Fabaceae/NA		Leaves	Antimicrobial activity and antidiarrheal effects	Ethiopia/[21]

*C. aurea* (Ait.)/Benth./Fabaceae/NA	Human: rheumatism	NA		Ethiopia/[32]

*C. aurea* (Ait.) Benth./Fabaceae/2008	Human: antidiarrheal	Leaves		Ethiopia/[36]

*C. aurea* (Ait.) Benth./Fabaceae/AN6	Veterinary: antiparasitic (internal and external parasite)	Leaves		Ethiopia/[39]

*C. aurea* (Ait.) Benth./Fabaceae/ermiasLX76	Veterinary: tick infestation, helminthiasis, snake bite (root), and sore and parasitic leech (bulb)	Leaves, root and bulb		Ethiopia/[37]

*C. aurea* (Ait.) Benth./Fabaceae/YY03	Veterinary: black leg, ectoparasites	Leaves		Ethiopia/[38]

*C. aurea* (Ait.) Benth./Fabaceae/NA		Seeds	Antihypertensive activity	Ethiopia/[41]

Fabaceae/*C. aurea* (Ait.) Benth./*N. Crouch 1279*, NH		Stem and bark	Anticancer activity	South Africa/[42]

Fabaceae/*C. aurea* (Ait.) Benth./Fabaceae/Aded Med 2007/1–10		Leaves and stems	Antibacterial and antioxidant	South Africa/[17]

*C. macrostachyus *Del./Euphorbiaceae/AN59	Veterinary: diarrhea (dysentery), external parasite	Leaves		Ethiopia/[39]

*C. macrostachyus *Del./Euphorbiaceae/YY10	Bloat	Leaves		Ethiopia/[38]

*C. macrostachyus *Del./Euphorbiaceae/ermiasLX17	Veterinary: ringworm, dermatophilosis, mange, sore, scabies, and wound	Leaves		Ethiopia/[37]

*C. macrostachyus *Hochst. ex. Delile/Euphorbiaceae/2007	Human: skin rash and dandruff	Bud		Ethiopia/[36]

*C. macrostachyus *Del./Euphorbiaceae/AHU158	Human: liver disease/jaundice	Bark		Ethiopia/[35]

*Croton macrostachys*/Euphorbiaceae/NA		Leaves	Antibacterial activity	Cameroon/[25]

*Croton macrostachyus*/Euphorbiaceae		Stem bark	Antimicrobial activity	Kenya/[24]

*C. macrostachyus *Del./Euphorbiaceae/NA		NA	Antibacterial activity	Kenya/[18]

*C. macrostachyus *Del./Euphorbiaceae/NA	Human: rheumatism	NA		Ethiopia/[32]

*C. macrostachyus *H. ex. Del./Euphorbiaceae/NA		Leaves	Antidiarrheal activity	Ethiopia/[43]

*C. macrostachyus *H./Euphorbiaceae/NA		Leaves	Antimalarial activity	Ethiopia/[22]

*C. macrostachyus *Del./Euphorbiaceae/NA		Leaves	Insecticidal: third instar larvae of *Anopheles arabiensis*	Ethiopia/[44]

*W. somnifera *(L.) Dun./Solanaceae/ermiasLX206	Veterinary: blackleg	Root		Ethiopia/[22]

*W. somnifera *(L.) Dun./Solanaceae/AHU141	Human: extended flow of menstruation/menometrorrhagia (bark & leaf), gallstone (root & leaf), and evil eye (branch)	Bark, branch, and leaves		Ethiopia/[37]

*W. somnifera *(L.) Dun./Solanaceae/NA		Leaf and root	Hypoglycaemic and hypolipidemic effects	India/[45]

*W. somnifera *(L.) Dun./Solanaceae/WS13		Leaf, fruit, and root	antioxidant properties and antibacterial activities	Bangladesh/[19]

*W. somnifera *(L.) Dunal./Solanaceae/NA	Veterinary: anthelmintic	NA		

*W. somnifera *(L.) Dunal./Solanaceae/NA		Stem	Antimicrobial	India/[23]

*W. somnifera *(L.) Dunal./Solanaceae/AN4	Veterinary: most diseases especially anthrax and 3-day sickness	Root		Ethiopia/[39]

*Nicotiana tabacum (N. tabacum)* L./Solanaceae/AN9	Veterinary: leech	Leaves		Ethiopia/[39]

*N. tabacum *L./Solanaceae/NA	Veterinary: anthelmintic	NA		Ethiopia/[34]

*N. tabacum *L./Solanaceae/2029	Veterinary and human: repels snakes from garden	NA		Ethiopia/[36]

*Nicotiana tabacum *L./Solanaceae/YY13	Veterinary: blackleg	Leaves		Ethiopia/[38]

*Nicotiana tabacum *L./Solanaceae/CVM57	Mastitis, ectoparasites and other skin diseases, and leech infestation	Leaves	Antimicrobial activity	Ethiopia/[20]

*E. brucei *Schwein./Fabaceae/AN12	Veterinary: internal and external parasites	Leaves		Ethiopia/[39]

NA: Not available.

**Table 2:** Percentage yields of plant extracts using methanol and chloroform.

Plant species	Solvent	Plant part used	Weight of sample (g)	Dry weight of extract (g)	Yield (%)
*C. macrostachyus *Del.	Methanol	Leaf	100	16.16	16.16%
	Chloroform	Leaf	100	18.33	18.33%

*C. aurea *(Ait.) Benth.	Methanol	Leaf	100	17.33	17.33%
	Chloroform	Leaf	100	10	10%

*W. somnifera *(L.) Dunal	Methanol	Root	100	14.16	14.16%
	Chloroform	Root	100	21.3	21.3%

*E. brucei *Schwein.	Methanol	Leaf	100	11.6	11.6%
	Chloroform	Leaf	100	16.66	16.66%

*N. tabacum *L.	Methanol	Leaf	100	9.3	9.3%
	Chloroform	Leaf	100	13.3	13.3%

**Table 3:** The antibacterial activity of methanol extracts of five medicinal plants zone of inhibition in diameter (mm).

Plant species used	Concentration (mg/ml)	Mean zone of inhibition in diameter (mm)		
		*S. aureus*	*E. coli*	*P. aeruginosa*
*C. aurea *(Ait.) Benth.	200	4.66^a,b^	4.03^d–f^	4.4^b–d^
	100	3.66^f,g^	3.3^g–i^	3.43^g,h^
	50	2.53^l,m^	2.66^k–m^	2.8^j–l^

*C. macrostachyus *Del.	200	4.43^b,c^	4.13^c–e^	4.2^c,d,e^
	100	2.86^j–l^	3.0^i–k^	2.86^j–l^
	50	2.0^o,p^	1.8^p,q^	2.33^m–o^

*W. somnifera *(L.) Dunal	200	3.96^e,f^	4.03^d–f^	4.26^c–e^
	100	2.4^m,n^	2.06^n–p^	2.33^m–o^
	50	1.7^p,q^	1.7^p,q^	1.7^p,q^

*N. tabacum *L.	200	3.1^h–j^	3.33^g–i^	2.53^l,m^
	100	2.86^j–l^	2.8^j–l^	2.86^j–l^
	50	1.8^p,q^	1.7^p,q^	1.6^q^

*E. brucei *Schwein.	200	0^r^	0^r^	0^r^
	100	0^r^	0^r^	0^r^
	50	0^r^	0^r^	0^r^

Streptomycin	200	4.9^a^	4.9^a^	4.9^a^
	100	4.9^a^	4.9^a^	4.9^a^
	50	4.9^a^	4.9^a^	4.9^a^

DMSO	200	0^r^	0^r^	0^r^
	100	0^r^	0^r^	0^r^
	50	0^r^	0^r^	0^r^

NB: levels not connected by the same letter are significantly different.

**Table 4:** The antibacterial activity of chloroform extracts of five medicinal plants zone of inhibition in diameter (mm).

Plant species used	Concentration (mg/ml)	Mean zone of inhibition in diameter (mm)		
		*S. aureus*	*E. coli*	*P. aeruginosa*
*C. aurea *(Ait.) Benth.	200	3.96^b–d^	4.0^b,c^	4.26^b^
	100	3.5^f^	2.33^l–p^	3.53^e,f^
	50	2.2^n–r^	2.26^m–q^	2.4^k–p^

*C. macrostachyus *Del.	200	3.93^b–e^	3.56^d–f^	3.73^c–f^
	100	3.0^g–i^	3.0^g–i^	3.03^g,h^
	50	2.13^o–r^	2.2^n–r^	2.4^k–p^

*W. somnifera *(L.) Dunal	200	3.4^f,g^	3.06^g,h^	2.6^i–n^
	100	2.86^h–j^	2.26^m–q^	2.4^k–p^
	50	1.8^r,s^	2.0^p–s^	2.0^p–s^

*N. tabacum *L.	200	2.66^h–m^	2.8^h–k^	2.53^j–o^
	100	2.73^h–l^	2.46^j–o^	2.4^k–p^
	50	1.6^s^	1.8^r,s^	1.9^q–s^

*E. brucei *Schwein.	200	0^t^	0^t^	0^t^
	100	0^t^	0^t^	0^t^
	50	0^t^	0^t^	0^t^

Streptomycin	200	4.9^a^	4.9^a^	4.9^a^
	100	4.9^a^	4.9^a^	4.9^a^
	50	4.9^a^	4.9^a^	4.9^a^

DMSO	200	0^t^	0^t^	0^t^
	100	0^t^	0^t^	0^t^
	50	0^t^	0^t^	0^t^

NB: levels not connected by the same letter are significantly different.

## Additional Points

*Availability of Data and Materials.* All data generated during this study are included in this published article. Plant materials used in this study have been identified at the National Herbarium of Addis Ababa University and voucher specimens are deposited at the College of Agriculture and Natural Resource, Dilla University. The bacterial strains used for the experiment are available at the Ethiopian public health institute.
